# Comprehensive Analysis of Shear Deformation Cytometry Based on Numerical Simulation Method

**DOI:** 10.3390/bios15060389

**Published:** 2025-06-17

**Authors:** Jun Wang, Jiahe Chen, Wenlai Tang, Shu Zhu

**Affiliations:** 1 Wenzhou Key Laboratory of AI Agents for Agriculture, Wenzhou Academy of Agricultural Sciences, Wenzhou 325006, China; 2 Jiangsu Key Laboratory of 3D Printing Equipment and Manufacturing, School of Electrical and Automation Engineering, Nanjing Normal University, Nanjing 210023, China; 3 University of Glasgow, Glasgow G12 8QQ, UK; 4 Nanjing Institute of Intelligent High-End Equipment Industry Co., Ltd., Nanjing 210042, China

**Keywords:** cell deformation, numerical simulation, shape changes, microfluidics

## Abstract

The deformability of cells reflects their capacity for shape changes under external forces; however, the systematic investigation of deformation-influencing factors remains conspicuously underdeveloped. In this work, by using an incompressible neo-Hookean viscoelastic solid model, coupled with the Kelvin–Voigt model, the effects of flow rate, fluid viscosity, cell diameter, and shear modulus on cell deformability were systematically calculated and simulated. Additionally, the relationship between cell deformability and relaxation time within a dissipative process was also simulated. The results indicate that cell deformation is positively correlated with flow rate, with an approximate linear relationship between the deformation index and flow velocity. Fluid viscosity also significantly affects cell deformation, as an approximate linear relationship with the deformation index is observed. Cell diameter has a more prominent impact on cell deformability than do flow rate or fluid viscosity, with the deformation index increasing more rapidly than the cell diameter. As the Young’s modulus increases, cell deformation decreases non-linearly. Cell deformation in the channel also gradually decreases with the increase in relaxation time. These findings enhance the understanding of cell biophysical characteristics and provide a basis for the precise control of cell deformation in deformability cytometry. This research holds significant implications for cell analysis-based animal health monitoring in the field of agriculture, as well as for other related areas.

## 1. Introduction

Cell deformability, a fundamental biophysical characteristic, is intricately linked to the physiological and pathological states of cells [1,2,3]. In the context of agriculture, understanding the factors that influence cell deformability holds great promise for addressing various challenges [4,5], enhancing animal health [6,7], and developing effective pest control strategies [7,8].

In animal husbandry, cell deformability, especially that of blood cells, serves as a valuable indicator of animal health [9]. Red blood cells, for example, must be highly deformable to pass through the narrow capillaries and deliver oxygen to tissues [10]. Any alteration in their deformability can lead to impaired blood flow and oxygen delivery [11], which is often associated with various diseases. For example, in cases of anemia, the reduced deformability of red blood cells can cause them to clog the small blood vessels, leading to tissue hypoxia [12]. By analyzing the deformability of blood cells, veterinarians can detect early signs of diseases, monitor disease progression, and evaluate the effectiveness of treatments [13]. This can significantly improve the overall health and productivity of livestock. Moreover, in the study of plant–pest interactions, the deformability of insect cells is of great importance. Insects rely on the deformability of their cells to penetrate plant tissues, evade plant defense mechanisms, and establish successful infestations [14]. Understanding the factors that affect insect cell deformability can provide new insights into the development of novel pest control strategies [15]. For example, if we can identify the factors that cause insect cells to become less deformable, we may be able to develop biopesticides or genetic engineering approaches to target these factors and prevent insect pests from causing damage to crops.

Despite the significant importance of cell deformability in agriculture, the factors that affect cell deformation in cytometry have not yet been systematically investigated [16,17]. Although some works have conducted in-depth exploration of the factors affecting cell deformation, they have often limited themselves to exploring the impact of individual factors [18,19]. Previous research has mainly focused on specific cell types and a limited number of influencing factors [20]. For blood cells, which have a relatively simple structure, several modeling methods have been developed [21]. The immersed boundary method is a widely used approach for modeling blood cells [22,23]. It can effectively capture the elasticity, surface tension, and bending stiffness of the cell membrane, allowing researchers to simulate the behavior of blood cells in blood flow. Another commonly used method is dissipative particle dynamics [19]. Early applications of this method considered the cellular elasticity of red blood cells and simulated them as elastic solids. In recent years, multi-particle collision dynamics models have gained popularity in the study of blood cell dynamics [24,25]. These models describe cells as elastic capsules with bending stiffness, providing a more realistic representation of the mechanical behavior of cells. Although the immersive boundary method can effectively simulate the elasticity, surface tension, and bending stiffness of cell membranes, it falls short in dealing with complex fluid environments and multicellular interactions. The dissipative particle dynamics and multi-particle collision dynamics models focus more on the single property of cells as elastic solids or elastic capsules, ignoring the comprehensive influence of dynamic changes in internal structure and external environmental factors on cell deformability. Furthermore, these existing studies lack a systematic understanding of the factors influencing cell deformability in cytometry [26,27], which has limited the development of accurate and efficient methods for cell analysis in agriculture.

The primary objective of this study is to conduct a comprehensive analysis of shear deformation cytometry using numerical simulation methods to systematically investigate the effects of multiple factors on cell deformability. Owing to the fact that the detection of the leukocyte subpopulation is of great significance in the diagnosis of animal diseases, the main research object of our work is the leukocyte subpopulation. In this work, we aim to study the influence of flow rate, fluid viscosity, cell diameter, and shear modulus on the deformability of cells. Additionally, the relationship between cell deformability and relaxation time within a dissipative process is also studied. To achieve our research objectives, we will use an incompressible neo-Hookean viscoelastic solid model coupled with the Kelvin–Voigt model. This coupled model is designed to more accurately represent the viscoelastic properties of real cells, allowing us to observe the stress and strain distributions when cells enter the channel of a deformation cytometry system. We will employ simulation software to replicate the forces acting on the cells and their deformation under different conditions. By carefully setting the boundary conditions, mesh parameters, and governing equations, we will ensure the accuracy and reliability of our simulations. The significance of this research is multi-fold. Firstly, it will enhance our understanding of the biophysical characteristics of cells by providing a more comprehensive picture of the factors influencing cell deformability. This knowledge can help researchers in the field of agriculture to better understand the underlying mechanisms of animal health and plant–pest interactions. Secondly, our findings will provide a basis for the precise control of cell deformation in deformability cytometry. This can lead to the development of more accurate and efficient cell analysis techniques, which can be applied in animal health monitoring and pest control. In conclusion, this study represents a significant step forward in the field of cell deformability research in agriculture. By systematically investigating the factors influencing cell deformation, we hope to contribute to the development of innovative solutions that can address some of the most pressing challenges in modern agriculture.

## 2. Methods

### 2.1. Modeling and Numerical Simulation of Shear Deformation Cytometry

In this work, the forces acting on the cells and their subsequent deformation were simulated using simulation software (COMSOL Multiphysics 5.6), replicating different conditions such as various flow rates, fluid viscosities, cell diameters, cell viscosities, shear moduli, and relaxation times.

Due to the viscoelastic properties of cells, this work utilized an incompressible neo-Hookean viscoelastic solid to simulate biological cells and coupled them with the Kelvin–Voigt model to make the sample more in line with the physical properties of the real cells, thereby observing the stress and strain distributions encountered when cells enter into the channel of deformation cytometry. The inlet boundary condition was set to a fully developed flow, and the outlet was chosen to have a static pressure of 0 Pa. Moreover, the velocity distribution of the channel and the stress distribution of the cells under pressures of 0 Pa and high vacuum conditions were compared (Figure S1). It was found that there is no significant difference in the simulated velocity distribution of the channel and the simulated stress distribution of the cells between the setting pressures of 0 Pa and 10^−7^ Pa. Therefore, setting the static pressure at the outlet to 0 is reasonable. When the height and width are similar in a commonly used microchannel, the two-dimensional model can capture the main deformation characteristics of the three-dimensional model. Compared to the three-dimensional model, the two-dimensional model could maintain its accuracy when simulating the stress and strain of cells in the square microfluidic channel, reducing the complexity of simulation calculation. To better simulate cell deformation, such as that of the leukocytes in animal blood, a circular shape was chosen for the original shape of the cell model. To ensure the feasibility of the simulation, the flow rate [28], viscosity [29], Young’s modulus [2], and size [26] of the simulated cells were all selected based on the parameters used in reported works. According to the cellular properties of animal cells, the cell size range in this simulation model was 8–16 μm, the cell viscosity range was 1–5 mPa·s, and the cell Young’s modulus range was 1500–7500 Pa.

In order to make the model more realistic, the full coupling mode was selected for the fluid–solid coupling module. The mesh selected was an extremely refined free triangle mesh, and the resolution of the narrow region, the maximum unit growth rate, the maximum and minimum unit size, the boundary layer attributes, and other important parameters were set to reasonable levels to guarantee that the mesh transitioned smoothly and to ensure the accuracy of the calculation. Specifically, the maximum and minimum element sizes of the grid were 0.67 μm and 0.002 μm, respectively. The maximum element growth rate was 1.05. The narrow area resolution was 1. The number of layers in the boundary layer was 2, the stretching factor was 1.2, and the thickness was adjusted to 5. The coupling algorithm was in fully coupled mode, the linear solver employed was MUMPS, and the nonlinear method was automatic. The termination technique was tolerance, with a maximum iteration count of 4 and a tolerance factor of 1. The fully coupled mode can synchronously solve multiple interacting physical fields (fluids, solids) through a unified system of equations, rather than requiring their independent calculation in steps. Its core advantage lies in the precise capture and efficient processing of the coupling effect of multiple physical field strengths, especially suitable for scenarios in which there is significant dynamic interaction between fields.

**Figure 1:**
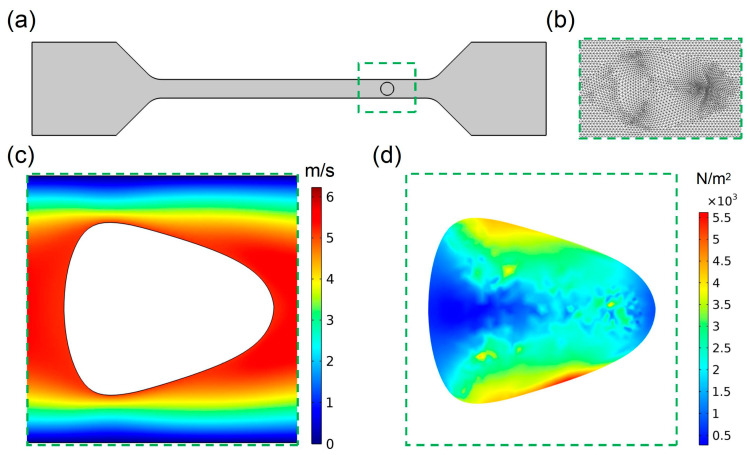
(**a**) Planar structure of the model; (**b**) mesh division of the model; (**c**) flow velocity distribution of the fluid around the cell in the channel; (**d**) stress distribution of the cell in the channel.

Figure 1a shows the schematic structure of the model; the deformation cytometry in this work was mainly composed of a straight channel with cross section measuring 20 μm × 20 μm. To improve the accuracy of the simulation as much as possible, the mesh was carefully dissected, and Figure 1b shows the results of the mesh dissection near the cell at a certain moment during the calculation process. To verify the feasibility of our model, the velocity and stress distribution in the channel and the cell were simulated preliminarily. From the velocity distribution (Figure 1c) and the stress distribution (Figure 1d) suffered by the cell in the channel at the same moment, it could be seen that the cell can generate stable deformation in the channel of deformation cytometry.

### 2.2. Governing Equations for Numerical Simulation

The flow of liquid in the channel was governed by velocity field **u** for the spatial moving coordinate system, and the incompressible Navier–Stokes equation for liquid pressure *p*; specific formulas can be described as follows:

(1) $$u=\left(µ,\;v\right)$$

(2) $$\rho \frac{\partial u}{\partial t}-\left[-\rho I+\eta \left(\nabla u+{\left(\nabla u\right)}^{T}\right)\right]+\rho \left(\left(u-u_{m}\right)\cdot \nabla \right)u=F-\nabla \cdot u$$

In Formula (1), **u** represents the velocity vector of a fluid at each point in the plane; µ and v represent the components of the velocity in the *x*-axis and *y*-axis directions, respectively. In Formula (2), I represents unit diagonal matrix; η is the fluid viscosity; F represents the volumetric force that affects the fluid, assuming there is no gravity or other volumetric force affecting the fluid; thus, F=0; $u_{m}$ represents the velocity of the coordinate system.

The deformation of the hyperelastic materials is solved using nonlinear geometric formulas. The boundary of the hyperelastic materials bears the load applied by the fluid, and the formula is as follows:

(3) $$F_{T}=-n\cdot \left(-pI+\eta \left(\nabla u+{\left(\nabla u\right)}^{T}\right)\right)$$

In Formula (3), n is the normal vector of the boundary; this load ($F_{T}$) represents the sum of pressure and viscous force.

The cells in the shear deformation are mainly deformed by shear force. Shear force can be calculated as the shear stress acting on the cell, multiplied by half of cell area, and the formula for shear stress τ calculations is as follows:

(4) τ=γ∗η

In Formula (4), γ is the shear rate, and it can be calculated as $\gamma =\left(V_{1}-V_{0}\right)/a_{p}$, where $V_{1}$ is the flow velocity in constriction channel, $V_{0}$ is the flow velocity of the stationary cell, and $a_{p}$ is the diameter of the cell.

## 3. Results

### 3.1. The Influence of Flow Rate on Cell Deformation

According to published works and theories [30,31], it is obvious that the cellular deformation and the force on the cells are positively correlated with the velocity of fluids in the microchannel. In the coupled model of our work, the influence of flow rate on cell deformations was verified.

**Figure 2:**
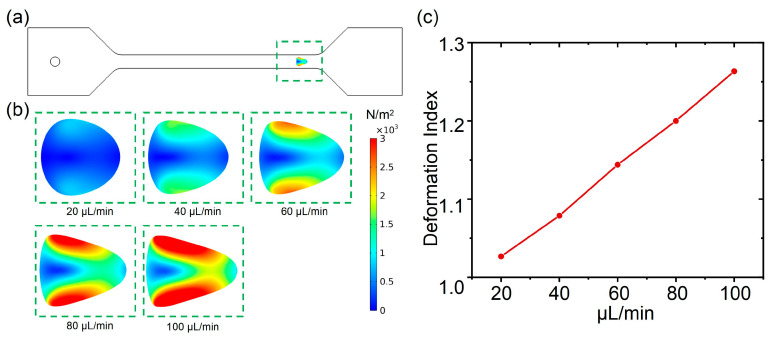
(**a**) Deformation of cells in the microchannel; (**b**) deformability of cells in flow rates ranging from 20 μL/min to 100 μL/min; (**c**) deformation index of cells under flow rates of 20~100 μL/min.

In this section, the deformability of cells in flow rates of 20~100 μL/min (with an interval of 20 μL/min) were calculated and simulated. From Figure 2a, the deformation of the cell was successfully observed when the cell was passing through the microchannel. From Figure 2b, it can be observed that when the flow rate increases, the deformation of the cell becomes greater. Significantly, with the increases in flow rate, the cell becomes flattened, and the flattened cell is gradually elongated. Furthermore, owing to the fact that the shear stress acts on the cell along the direction of the channel, the deformation of the cell on both sides along the channel direction is more pronounced. In contrast, the deformation on both sides of the cell, perpendicular to the flow channel direction, is relatively small, which may be attributed to less shear stress in this direction. Owing to the different deformations of cells in different directions, with the increases in flow rate, the shape of cell in the plane gradually changed from an ideal circle to an approximate triangle. Moreover, research on the process of cell shape changes is helpful for obtaining cellular bio-physical features and for cell type discrimination. In order to quantify the cell deformation, the deformation index under flow rates of 20~100 μL/min were calculated. From Figure 2c, it can be observed that with the increase in flow rate, the deformation index of the cell was increased as well, where the deformation index is the ratio between the long and short axes of the deformed cells (Figure S2). In addition, an approximate linear relationship between the deformation index and the flow velocity was observed. Therefore, by precisely manipulating the flow rate, the accurate control of cell deformation in deformability cytometry is expected to be achieved.

From the above results, it was observed that the flow rate of the fluid had significant impact on cell deformation. Although the manipulation of cell deformation by flow rate has been proven to display the advantages allowing for precise and easy operations, its limitations are still unignorable. To generate adequate cell deformation, a sufficiently high flow rate is necessary in the microchannel. However, a high flow rate in micrometer-scale channels tends to result in leakage, while the higher pressure required for higher flow rates may cause damage to the membranes of the cells. Furthermore, high cell velocity in the microchannel may cause difficulties in capturing images illustrating the cell deformation process, resulting in extremely high requirements for the hardware used in deformability cytometry.

### 3.2. The Influence of Fluid Viscosity on Cell Deformation

**Figure 3:**
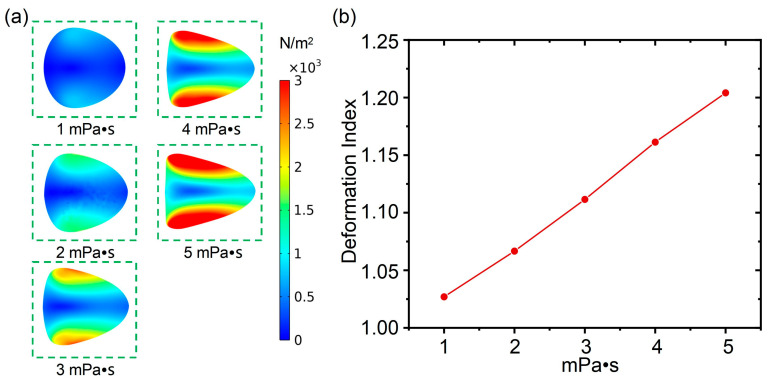
(**a**) Deformability of cells with fluid viscosity increasing from 1 mPa·s to 5 mPa·s; (**b**) deformation index of cell with fluid viscosity increasing from 1 mPa·s to 5 mPa·s.

According to the shear stress formula (Formula (4)), the magnitude of shear stress is also related to the viscosity of the fluid. Subsequently, the influence of fluid viscosity on cell deformability was explored. In this section, the deformability of cells under a low flow rate (20 μL/min) but a different fluid viscosity was calculated and simulated. As shown in Figure 3a, different cell deformations were observed when the fluid viscosity increased from 1 mPa·s to 5 mPa·s. Obviously, with the increase in fluid viscosity, the deformation of the cell gradually increased as well. Similar to the influence of flow rate on cell deformation, the shape of the cell in the plane gradually changed from an ideal circle to an approximate triangle with the increases in fluid viscosity. Moreover, the shape changes in the cells caused by fluid viscosity variations were also similar to those caused by differing flow rates. For example, the shear stress acting on the shape of the cell under a flow rate of 20 μL/min and a fluid viscosity of 4 mPa·s was nearly consistent with noted under a flow rate of 80 μL/min in the aforementioned section. Furthermore, to quantify the influence of fluid viscosity on cell deformation, the deformation index was also calculated. From Figure 3b, with the increases in fluid viscosity, the deformation index of the cell was gradually increased as well. Notably, the deformation index was over 1.20 under a flow rate of 20 μL/min and a fluid viscosity of 4 mPa·s, which is close to the deformation index calculated under a flow rate of 100 μL/min and a fluid viscosity of 1 mPa·s. Additionally, the approximate linear relationship between fluid viscosity and the deformation index was also observed. These results demonstrated the inference derived from the formula regarding shear stress, which is that the cell deformation can be manipulated by controlling the viscosity of the fluid when the flow rate is limited.

Owing to the disadvantages of cell deformation under a high flow rate discussed in the aforementioned text, significant cell deformation under a relatively low flow rate is meaningful. According to the influence of fluid viscosity on cell deformation, it was found that increasing the fluid viscosity can also result in sufficient cell deformation, even under a relatively low flow rate. The deformability of cells under variable flow rates and fluid viscosities is similar.

### 3.3. The Influence of Cell Diameter on Deformability

In deformability cytometry, the microchannel is typically narrow; thus, the diameter of the cell may have a significant influence on its deformability. For example, under conditions with a determined flow rate and fluid viscosity, the cell with dimensions close to the cross-sectional size of the channel may display higher deformability than that of a cell with smaller size.

**Figure 4:**
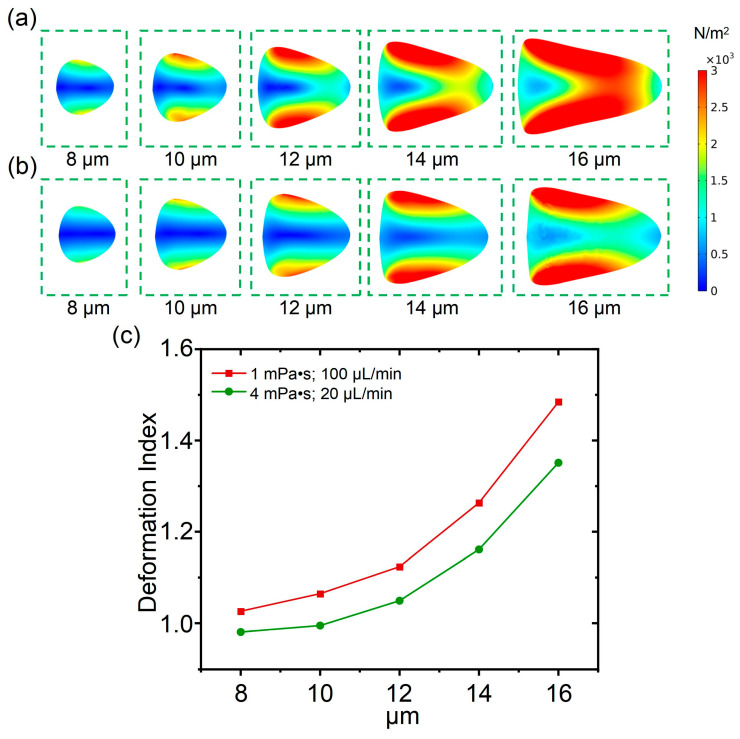
(**a**) The influence of cell diameter on deformability under a fluid viscosity of 1 mPa·s and a flow rate of 100 μL/min; (**b**) the influence of cell diameter on deformability under a fluid viscosity of 4 mPa·s and a flow rate of 20 μL/min; (**c**) cell deformation index when the cell diameter increases from 8 μm to 16 μm.

In this section, the influence of cell diameter on cell deformability is analyzed. As shown in Figure 4a, under the condition with a fluid viscosity of 1 mPa·s and a flow rate of 100 μL/min, the cell deformability was calculated with the cell diameter ranging from 8 μm to 16 μm (with an interval of 2 μm), which was used to simulate the leukocyte subpopulation in animal blood cells, such as neutrophils and lymphocytes. From Figure 4b, under the condition with a fluid viscosity of 4 mPa·s and a flow rate of 20 μL/min, the cell deformability was also calculated when the cell diameter ranged from 8 μm to 16 μm (with an interval of 2 μm). It was found that the cells on both sides along the channel direction are subjected to significant shear stress, while the cell deformation increased with increasing of cell diameters. Under conditions with a determined flow rate and fluid viscosity, cells with larger diameters tended to be squeezed into an approximate triangle shape, and the cell aspect ratio was larger as well. Compared to the condition with a fluid viscosity of 4 mPa·s and a flow rate of 20 μL/min, the shear stress impacted the cells, and the cell deformation was larger under the condition with a fluid viscosity of 1 mPa·s and a flow rate of 100 μL/min. In addition, compared with cell deformation induced by a varied flow rate or fluid viscosity, it is apparent that the influence of cell diameter on cell deformability was more significant. Subsequently, the cell deformation index for cells with diameters ranging from 8 μm to 16 μm was calculated to quantify the specific cell deformability. From Figure 4c, it can be observed that the cell deformation under the condition of a fluid viscosity of 1 mPa·s and a flow rate of 100 μL/min was more significant than that of the condition with a fluid viscosity of 4 mPa·s and a flow rate of 20 μL/min, which is consistent with the observation in the distribution diagram of shear stress. Moreover, compared to the linear relationships between cell deformation and flow rate, or between cell deformation and fluid viscosity, the increasing rate of the deformation index is faster than the increases in cell diameter.

As a result, through the comprehensive analysis presented in this section, the significant influence of cell diameter on cell deformation was revealed. Therefore, in a deformation cytometry with a determined channel size, cells with different sizes may reveal different deformability. Therefore, reducing the influence of size on deformation is crucial when studying the common deformation characteristics of cells from the same species, but of different sizes.

### 3.4. The Influence of Young’s Modulus on Cell Deformation

**Figure 5:**
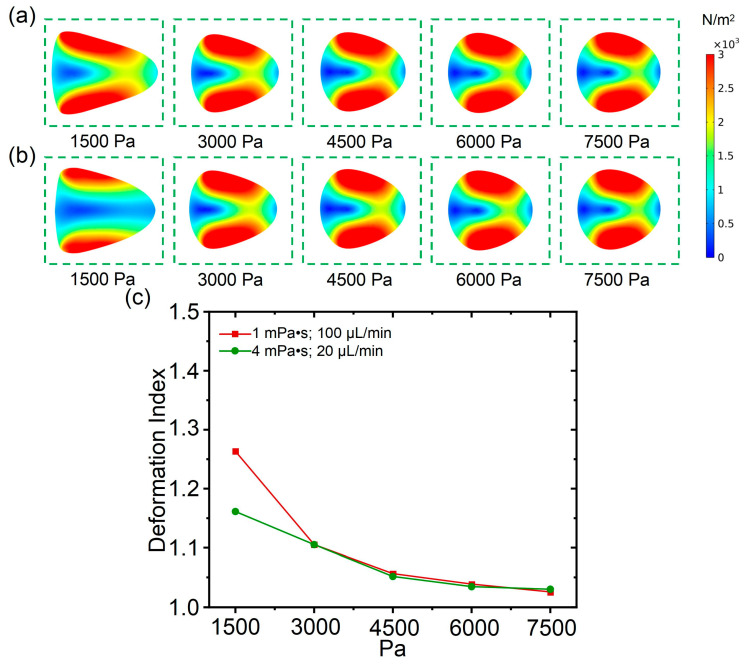
(**a**) The influence of cell Young’s modulus on deformability under a fluid viscosity of 1 mPa·s and a flow rate of 100 μL/min; (**b**) the influence of cell Young’s modulus on deformability under a fluid viscosity 4 mPa·s and a flow rate of 20 μL/min; (**c**) cell deformation index when cell Young’s modulus increases from 1500 Pa to 7500 Pa.

In deformation cytometry, the influence of the elasticity of cells on their deformability is crucial as well, and cells with different elasticities may exhibit differences in deformability. In this section, the influence of Young’s modulus on cell deformability was studied. From Figure 5a, it can be observed that with a fluid viscosity of 1 mPa·s and a flow rate of 100 μL/min, the cell deformability was calculated with a Young’s modulus ranging from 1500 Pa to 7500 Pa (with an interval of 1500 Pa). As shown in Figure 5b, under the condition with a fluid viscosity of 4 mPa·s and a flow rate of 20 μL/min, the cell deformability was calculated with the Young’s modulus ranging from 1500 Pa to 7500 Pa. According to the numerical simulation, with the increase in the cell’s Young’s modulus, the cell deformation gradually decreased, and the cell was changed from an approximate triangle shape to an approximate circle shape. From the planar shear stress distribution of the cell, the maximum and minimum values of shear stress for cells with different Young’s modulus values were almost equal. Therefore, even if the shear stress acting on the cells is consistent, the value of the cells’ Young’s modulus has a significant influence on cell shape changes. By comparing the simulation results with those shown in Figure 5a,b, it was found that the cell deformation with a fluid viscosity of 4 mPa·s and a flow rate of 20 μL/min was less than that with fluid viscosity of 1 mPa·s and a flow rate of 100 μL/min when the value of Young’s modulus is 1500 Pa, which indicates that the flow rate has a greater impact on cell deformation when the Young’s modulus of the cells is same.

Subsequently, to quantify the influence of Young’s modulus on cell deformability, a related deformation index was calculated. From Figure 5c, it can be observed that as the value of Young’s modulus increases, the cell deformation under conditions with a determined flow rate and fluid viscosity gradually decreased. The relationship between the cell deformation index and Young’s modulus was non-linear, and the decreasing rate of the deformation index is faster than the increases in the Young’s modulus. Furthermore, when the Young’s modulus displays a value of 1500 Pa, a significant difference in the cell deformation index between conditions, as illustrated in Figure 5a,b, was observed. When the Young’s modulus increases continuously, the cell deformation index between conditions, as illustrated in Figure 5a,b, were nearly overlapping. From this result, for cells with a relatively large Young’s modulus, it is easier to achieve similar deformation between conditions with different flow rates and fluid viscosities.

### 3.5. The Relationship Between Cell Deformability and Relaxation Time

Relaxation time refers to the characteristic time required for a system in a non-equilibrium state to gradually return to equilibrium through internal interactions after removing external disturbances [32,33]. In the research of cell deformability, relaxation time reflects the ability and time scale of cells to recover to their initial state or to reach a new stable state after being subjected to force deformation, and the research on the effect of relaxation time on cell deformation is meaningful.

**Figure 6:**
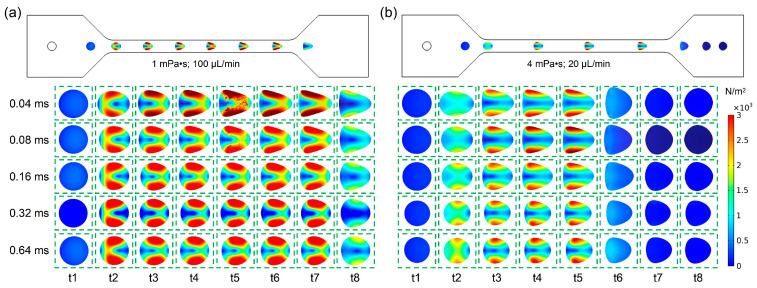
(**a**) The relationship between cell deformability and relaxation time under fluid viscosity of 1 mPa·s and flow rate of 100 μL/min; (**b**) The relationship between cell deformability and relaxation time under fluid viscosity 4 mPa·s and flow rate of 20 μL/min.

As shown in Figure 6a, under the condition with a fluid viscosity of 1 mPa·s and a flow rate of 100 μL/min, the cell deformability was calculated and simulated when the cell relaxation time ranged from 0.04 ms to 0.64 ms. From Figure 6b, it can be observed that under the condition with a fluid viscosity of 4 mPa·s and a flow rate of 20 μL/min, the cell deformability was calculated and simulated when the cell relaxation time ranged from 0.04 ms to 0.64 ms. To clearly display the influence of relaxation time on cell deformation, the cell deformation at eight time points (t1~t8) is displayed as the cell it passes through the channel of a deformation cytometry. By comparing the shear stress distribution, shown in Figure 6a,b, it was found that the cell deformation under the condition with a fluid viscosity of 1 mPa·s and a flow rate of 100 μL/min was larger than that noted under the condition with a fluid viscosity of 4 mPa·s and a flow rate of 20 μL/min. This result demonstrated that the influence of flow velocity on deformation is greater when the relaxation time was fixed. In addition, the cell deformation gradually increased when the cell entered the channel inlet, while the cell deformation gradually recovered when the cell entered the channel outlet. Owing to the difference in relaxation time, there were also differences in the times for the cells to reach their maximum deformation and the time for the cells to recover. Furthermore, with the increase in relaxation time, the cell deformation in the channel gradually decreased.

Therefore, according to the simulation results, the relationship between cell deformability and relaxation time as a dissipative process has been verified, which helps to acquire and research the biophysical characteristics of the cells.

## 4. Conclusions

In this work, to investigate the factors influencing cell deformation in deformability cytometry, a comprehensive analysis of shear deformation cytometry is carried out based on a numerical simulation method. To simulate the realistic deformation of cell as much as possible, an incompressible neo-Hookean viscoelastic solid model, coupled the Kelvin–Voigt model, is applied. Based on this coupled model, the effects of flow rate, fluid viscosity, cell diameter, and shear modulus of the cell on cell deformability are systematically calculated and simulated. Moreover, the relationship between cell deformability and relaxation time within a dissipative process is also studied. The results show that flow rate has a positive correlation with cell deformation, and an approximate linear relationship exists between the deformation index and flow velocity. Fluid viscosity also has a significant impact on cell deformation. By increasing fluid viscosity, sufficient cell deformation can be achieved, and there is an approximate linear relationship between fluid viscosity and the deformation index. Cell diameter has a more substantial influence on cell deformability compared to that of flow rate or fluid viscosity. The deformation index increases faster than the increase in cell diameter. Regarding Young’s modulus, as it increases, cell deformation decreases non-linearly. Additionally, relaxation time also greatly affects cell deformability. With the increase in relaxation time, cell deformation in the channel gradually decreases. These findings contribute to a better understanding of the biophysical characteristics of cells, providing a basis for the accurate control of cell deformation in deformability cytometry. Certainly, some limitations of this work are non-negligible. For example, the simulation results lack direct comparison with the experimental results. Therefore, more cellular experiments would be meaningful for promoting a simulation-based study on deformability cytometry. Considering the application of shear deformation cytometry in the field of agriculture, it is convinced that this work has a significant impact for cell analysis-based animal health monitoring, and for other fields as well.

## Data Availability

Detailed data can be obtained from the authors.

## Supplementary Materials

The following supporting information can be downloaded at: https://www.mdpi.com/article/10.3390/bios15060389/s1, Figure S1: (a) Flow velocity distribution of the fluid around the cell at an outlet pressure of 0 Pa. (b) Flow velocity distribution of the fluid around the cell at an outlet pressure of 0.0000001 Pa. (c) Stress distribution of the cell at an outlet pressure of 0 Pa. (d) Stress distribution of the cell at an outlet pressure of 0.0000001 Pa; Figure S2: Definition of deformation index. Deformation index = a/b, where a and b are the long and short axes of deformed cells, respectively.
