# Supplementary material for: Comprehensive Analysis of Shear Deformation Cytometry Based on Numerical Simulation Method
Source: Biosensors (Basel). 2025 Jun 17;15(6):389. doi: 10.3390/bios15060389 (PMC12190582; doi:10.3390/bios15060389)
Supplement: Supplementary file 1 [file biosensors-15-00389-s001.zip › biosensors-3619098-supplementary.pdf]

Comprehensive Analysis of Shear Deformation Cytometry Based on Numerical Simulation Method

Jun Wang [1], Jiahe Chen [3], Wenlai Tang [2,4,*] and Shu Zhu [2,*]

[1] Wenzhou Key Laboratory of AI Agents for Agriculture, Wenzhou Academy of Agricultural Sciences, Wenzhou 325006, China
[2] Jiangsu Key Laboratory of 3D Printing Equipment and Manufacturing, School of Electrical and Automation Engineering, Nanjing Normal University, Nanjing 210023, China
[3] University of Glasgow, Glasgow G12 8QQ, UK
[4] Nanjing Institute of Intelligent High-End Equipment Industry Co., Ltd., Nanjing 210042, China
* Correspondence: wltang@njnu.edu.cn (W.T.); shu.zhu@nnu.edu.cn (S.Z.)

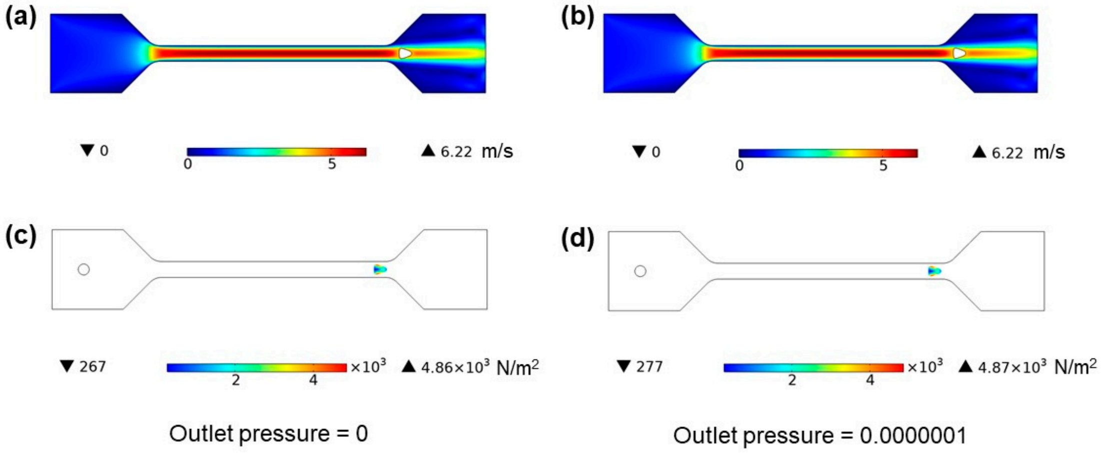

Figure S1. (a) Flow velocity distribution of the fluid around the cell at outlet pressure of 0 Pa. (b) Flow velocity distribution of the fluid around the cell at outlet pressure of 0.0000001 Pa. (c) Stress distribution of the cell at outlet pressure of 0 Pa. (d) Stress distribution of the cell at outlet pressure of 0.0000001 Pa.

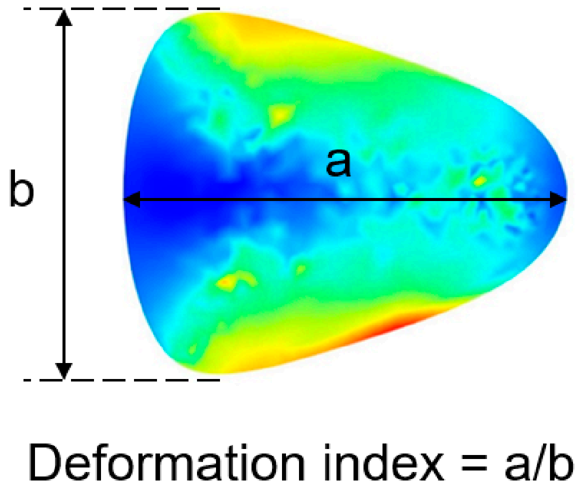

Figure S2. Definition of deformation index, deformation index = **a/b**, where the **a** and **b** are long and short axes of deformed cells, respectively.
